# External validation of an admission risk score for predicting inpatient paediatric mortality in two Kenyan public hospitals.

**DOI:** 10.12688/wellcomeopenres.23471.1

**Published:** 2024-12-24

**Authors:** Stephen Kamau, Joyce Kigo, Michuki Maina, John Gachohi

**Affiliations:** 1 Health Services Unit, KEMRI-Wellcome Trust Research Programme Nairobi, Nairobi, Kenya; 2 School of Public Health, Jomo Kenyatta University of Agriculture and Technology, Nairobi, Kenya; 3 Washington State University Global Health Program-Kenya, Nairobi, Kenya

**Keywords:** Children, risk score, external validation, admission, mortality, Kenya

## Abstract

**Background:**

Early identification of children at risk of mortality during hospitalization is crucial in preventing mortality in low-and middle-income countries (LMICs). This study aimed to externally validate an admission risk score for predicting inpatient paediatric mortality in resource-limited settings.

**Methods:**

This retrospective study utilized routine clinical data of children aged ≤12 years admitted to two Kenyan public hospitals between January 2017 and October 2023. The admission risk score includes 13 clinical predictors, each assigned a value. Aggregate values were used to predict inpatient pediatric mortality, with a higher score indicating a greater risk of death. Children with scores of 0, 1–4 and ≥5 were categorized as low, moderate and high-risk categories, respectively. Discrimination was assessed using area under the receiver operating characteristic curve (AUC). Sensitivity, specificity, and positive and negative predictive values were calculated at different cutoff points.

**Results:**

A total of 15,606 children were included in the study. Majority of the participants were male (8,847, 56.7%) and aged 12 – 59 months (7,222, 46.3%). Children classified as high-risk had a higher mortality rate (23.4%) than those classified as low risk (2%). The risk score demonstrated moderate discrimination, with an AUC of 0.73 (95% confidence interval (CI): 0.71 – 0.75). A cutoff of ≥3 achieved a balance between sensitivity and specificity, with values of 63.8% (95% CI: 60.7%–66.9%) and 72.2% (95% CI: 71.5% - 72.9%), respectively, compared to other cutoff points.

**Conclusion:**

The risk score performed moderately in predicting inpatient paediatric mortality in two Kenyan public hospitals. The risk score can be used with other clinical assessments to rapidly identify high-risk children and guide targeted interventions to prevent mortality.

## Plain language summary

Identifying children at high risk of mortality during hospitalization is essential, especially in low- and middle-income countries. Healthcare providers can utilize risk assessment tools to evaluate and identify paediatric patients at elevated risk of mortality upon admission to hospital wards. These risk stratification scores are valuable tools that integrate clinical examination findings, medical history data, and/or laboratory results to estimate the probability of specific outcomes, such as hospital admission, clinical deterioration, or death. These tools enable healthcare workers to prioritize high-risk patients and implement timely, life-saving interventions, thereby reducing morbidity and mortality rates.

In this study, we evaluated the performance of an admission risk score in predicting mortality among paediatric patients aged 12 years and below admitted to two public hospitals in Kenya. The risk assessment score demonstrated moderate ability in differentiating between children who survived and those who died during hospitalization. This risk stratification score can be employed in conjunction with other clinical assessments to prioritize care for paediatric patients at high risk of in-hospital mortality, potentially improving patient outcomes and resource allocation in resource-constrained settings.

## Introduction

The global burden of paediatric mortality remains high, with approximately 5 million deaths of children under five years old recorded in 2020 ^1^. Despite efforts to reduce paediatric mortality, the death rate of children admitted to hospitals in Sub-Saharan Africa remains high, with most deaths caused by preventable diseases such as diarrhoeal diseases, malaria, malnutrition, and pneumonia ^2– 5^. Furthermore, most deaths occur within the first 24 hours of admission ^6^. Hence, it is crucial to rapidly screen and identify children who are at risk of severe outcomes, including mortality, during hospitalization from all admitted patients and to prevent these deaths.

Risk scores, also known as clinical prediction rules (CPRs), risk stratification scores, clinical prediction models (CPMs), or decision rules, combine clinical examination, history, and/or diagnostic tests to stratify patients based on their probability of experiencing a target outcome ^7, 8^. Clinical signs and symptoms are assigned points, enabling prediction of the risk of an outcome and allowing the clinical team to identify high-risk patients. Although the majority of the paediatric risk scores developed have not been incorporated in clinical setting, some risk assessment tools, such as the Pediatric Risk of Mortality (PRISM) and Bedside Pediatric Early Warning System (Bedside PEWS), have been implemented in some hospitals and have proven instrumental in evaluating prognosis and detecting early signs of clinical deterioration among paediatric patients admitted to specialized units. By providing quantitative measures of patient status, these tools enable healthcare professionals to make informed decisions, allocate resources effectively, and initiate timely interventions, ultimately improving patient outcomes and the quality of care in paediatric settings ^9, 10^.

Several risk scores have been developed, but few have been validated in independent cohorts ^11, 12^. External validation of risk scores is recommended to avoid duplication and for the development of new risk scores for each hospital or region. Moreover, validation of risk scores helps assess their generalizability to different patient populations before implementation in clinical practice ^13^. Most of these risk scores have been developed in high-income countries, with only a few derived in low-resource settings. One such risk score developed in a resource-limited setting is the admission risk score by Mpimbaza *et al.*, which predicts all-cause mortality among hospitalized children ^14^. To the best of our knowledge, the performance of this risk score has not been externally validated elsewhere. The risk score must be validated in a different setting before incorporating it into clinical workflow. This study aimed to assess the performance of the admission risk score in predicting mortality among children aged 0–12 years admitted to two public hospitals located in two contrasting regions (rural and urban) in Kenya.

## Methods

### Reporting

This retrospective study adheres to the Transparent Reporting of a Multivariable Prediction Model for Individual Prognosis or Diagnosis (TRIPOD) guidelines for reporting clinical prediction models developed using machine learning or regression methods ^15, 16^.

### Study setting and data source

This study utilized routine clinical data from two County Referral Hospitals, between January 2017 and October 2023. These hospitals serve as referral centres for lower-level healthcare facilities within their respective counties. Both hospitals are part of the Clinical Information Network (CIN), which is a hospital surveillance platform that comprises the Kenya Medical Research Institute-Wellcome Trust (KWTRP), Kenya Ministry of Health (MOH), Kenya Paediatric Association (KPA) and participating hospitals. CIN aims to enhance patient care, improve data quality and reporting, and bolster inpatient paediatric surveillance data by improving the collection and utilization of routine data through systematic audit and feedback processes ^17^. CIN includes 24 hospitals distributed across 19 counties, providing comprehensive coverage of clinical admission details for children aged one month and above who are admitted to the paediatric wards. This extensive data collection system enables CIN to generate valuable insights into paediatric care practices, identify areas for improvement, and contribute to evidence-based decision-making in healthcare settings.

### Study population and eligibility

Children aged 12 years and below admitted to the paediatric wards of the two hospitals between January 2017 and October 2023 were eligible for inclusion in the study. Children missing hospital outcome, had more than 30% missing data on the predictors included in the admission risk score, or were admitted during the healthcare workers’ strike were excluded from the study.

### Data collection

Paediatric admission data is prospectively filled in a standardized paediatric admission record (PAR) by doctors, clinicians and nurses providing medical care to the children admitted to the paediatric wards of the study hospitals. PAR has been approved by the Ministry of Health and adopted by all hospitals participating in the CIN to improve documentation ^18^. The data include patient demographics, vital signs, history of illness, investigations, treatment received during the admission period, admission diagnosis and outcome at discharge. Trained data clerks abstract data from the PARs and enter them into a Research Electronic Data Capture (REDCap) tool ^19^. Data is then de-identified and synchronized to a central database based at the KWTRP office for storage and analysis. Data managers regularly carry out data quality monitoring visits to hospitals to authenticate the data. A summary of the variables included in the risk score and the corresponding variables in the paediatric REDCap is provided in Table 1.

**Table 1.** Summary of variables included in the admission risk score and corresponding variables in the database.

Variables included in the risk score	Corresponding variables in the REDCap database
Age ≤ 4 months	Age (months/years)
No subjective fever	Fever (Yes/ No)
Difficulty breathing	Difficulty breathing = (Yes/ No)
Altered consciousness	AVPU = Verbal or Pain response
Unconsciousness	AVPU = Unresponsive
Unable to drink/breastfeed	Can drink/breastfeed = (Yes/ No)
Convulsions	Convulsions = (Yes/ No)
Temperature ≤ 35.5°C	Temperature (continuous)
Pallor	Pallor = (Yes/ No)
Jaundice	Jaundice = (Yes/ No)
Deep breathing	Indrawing and acidotic breathing = (Yes/ No)
Unable to sit up or stand	Missing in the database
Signs of meningitis	Stiff neck and bulging fontanelle = (Yes/ No)

### Description of the admission risk score

The admission risk score is a prediction tool developed using prospectively collected data from a cohort of 50,249 children admitted to four public hospitals in Uganda ^14^. A logistic regression model was employed to identify 13 predictors of inpatient paediatric mortality, and the coefficients were converted into points for easier use in clinical settings. Age less than 4 months was found to have the highest odds of mortality and was assigned two points, while the remaining 12 predictor variables (no subjective fever, difficulty breathing, altered consciousness, unconsciousness, inability to drink or breastfeed, convulsions, temperature ≤ 35.5 °C, pallor, jaundice, deep breathing, inability to sit up or stand and signs of meningitis) were assigned one point in the final risk score. Patients who did not exhibit any of the signs or symptoms were assigned a score of zero, resulting in a composite risk score ranging from a minimum of zero to a possible maximum of 14 points. The cumulative score predicts inpatient paediatric mortality, with a higher score indicating an increased risk of death.

### Sample size

The sample size for external validation of the admission risk score was determined using the formula by Bujang *et al.*, 2018 ^20^. The minimum sample size for external validation was 750 paediatric records. However, this study utilized a larger sample size by including data for all eligible children.

The outcome was all-cause inpatient mortality.

### Missing data

To address missing data in the key variables used for risk score calculation, we implemented single imputation technique using the predictive mean matching (PMM) method within the Multiple Imputation by Chained Equations (MICE) package in R ^21^. We excluded samples with more than 30% missing values to ensure reliability of the imputation process. This approach was implemented to mitigate potential bias introduced by incomplete data sets. Single imputation offers a pragmatic solution for handling missing data in statistical analyses, particularly when the proportion of missing values is relatively low ^22^. By applying this method, we aimed to maximize the utilization of available data while maintaining the integrity of our risk score calculations.

### Statistical analysis

Categorical variables were summarized as proportions and percentages and continuous data were reported as median with inter-quartile range (IQR). The discriminatory ability of the risk score was assessed by plotting a receiver operating characteristic (ROC) curve and the associated area under the curve (AUC). The following cut-offs to categorize the discriminatory ability of the risk score were used: AUC <0.70 classified as "poor discrimination", 0.70 ≥ AUC ≤0.79 classified as "fair discrimination”, 0.80≥ AUC ≤0.89 classified as "good discrimination”, and AUC ≥0.90 classified as "excellent discrimination" ^23, 24^. Additionally, sensitivity, specificity, positive predictive value (PPV) and negative predictive value (NPV) of the risk score were calculated at different cut-off points. Children with cumulative score of zero, 1–4 and ≥ 5 were classified into low, moderate, and high-risk categories, respectively. The association between the risk categories and mortality was assessed using a chi-square test. All analyses were performed using an open-source R program, version 4.3.1 (R Foundation for Statistical Computing, Vienna, Austria, http://www.cran.r-project.org).

## Results

### Characteristics of the participants

**Figure 1.**
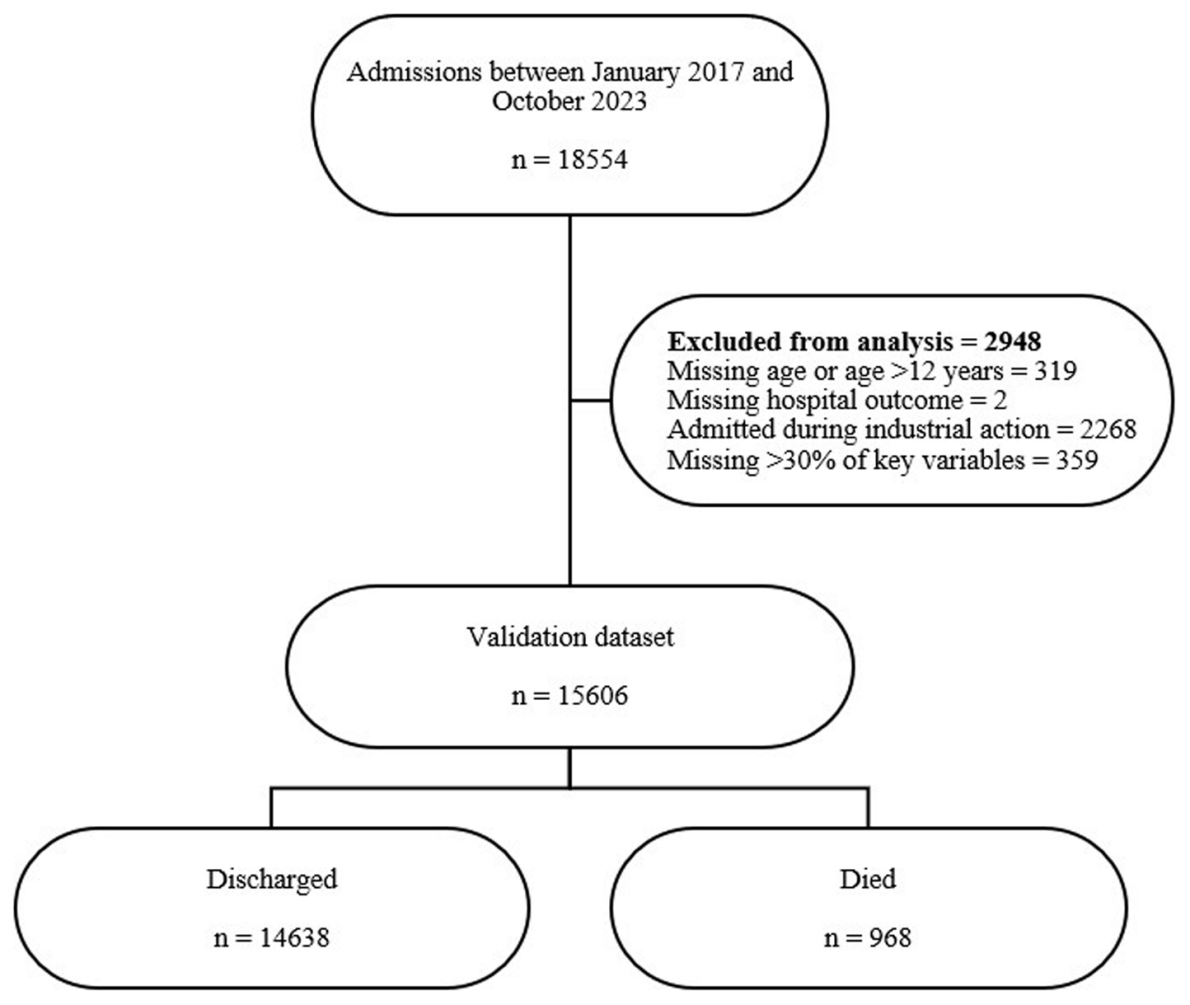
Flowchart of patients included in the analysis.

There were 18554 admissions between January 2017 and October 2023, of which 15606 (84.1%) were included in analysis ( Figure 1). Majority of the patients were male 8847 (56.7%). The median age was 14 months (IQR 7-34), and most children were aged between 12 and 59 months (7222, 46.3%). The median length of hospital stay was 6 days (IQR 3-9). The overall mortality rate during the study period was 6.2 % ( Table 2).

**Table 2.** Participants demographic characteristics.

Patient characteristics	Hospital		
	H1 n (%)	H2 n (%)	All n (%)
Total admitted	8754	6852	15606
Males	4888 (55.8%)	3959 (57.8%)	8847 (56.7%)
Age in months, median (IQR)	12 (6-28)	18 (8-36)	14 (7-34)
≤ 1 month	489 (5.6%)	298 (4.3%)	787 (5%)
2 – 11 months	3544 (40.5%)	2073 (30.3%)	5617 (36%)
12 – 59 months	3595 (41.1%)	3627 (52.9%)	7222 (46.3%)
60 - 144 months	1126 (12.9%)	854 (12.5%)	1980 (12.7%)
Hospital stay in days, median (IQR)	6 (4-9)	6 (2-8)	6 (3-9)
**Hospital outcome**			
Discharged alive	8012 (91.5%)	6626 (96.7%)	14638 (93.8%)
Died	742 (8.5%)	226 (3.3%)	968 (6.2%)

### Performance of the admission risk score

**Figure 2.**
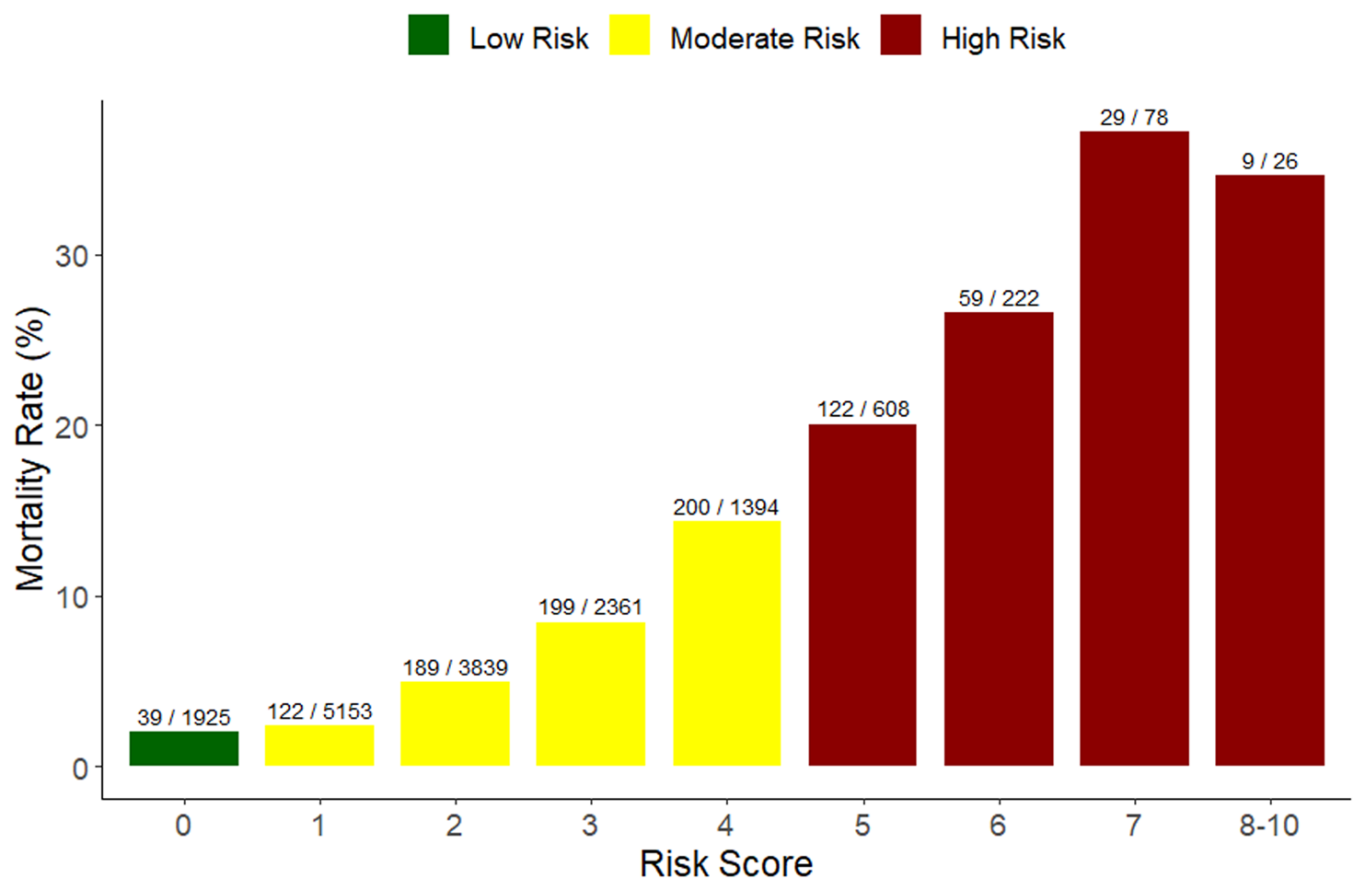
Inpatient paediatric mortality rate by risk score.

**Figure 3.**
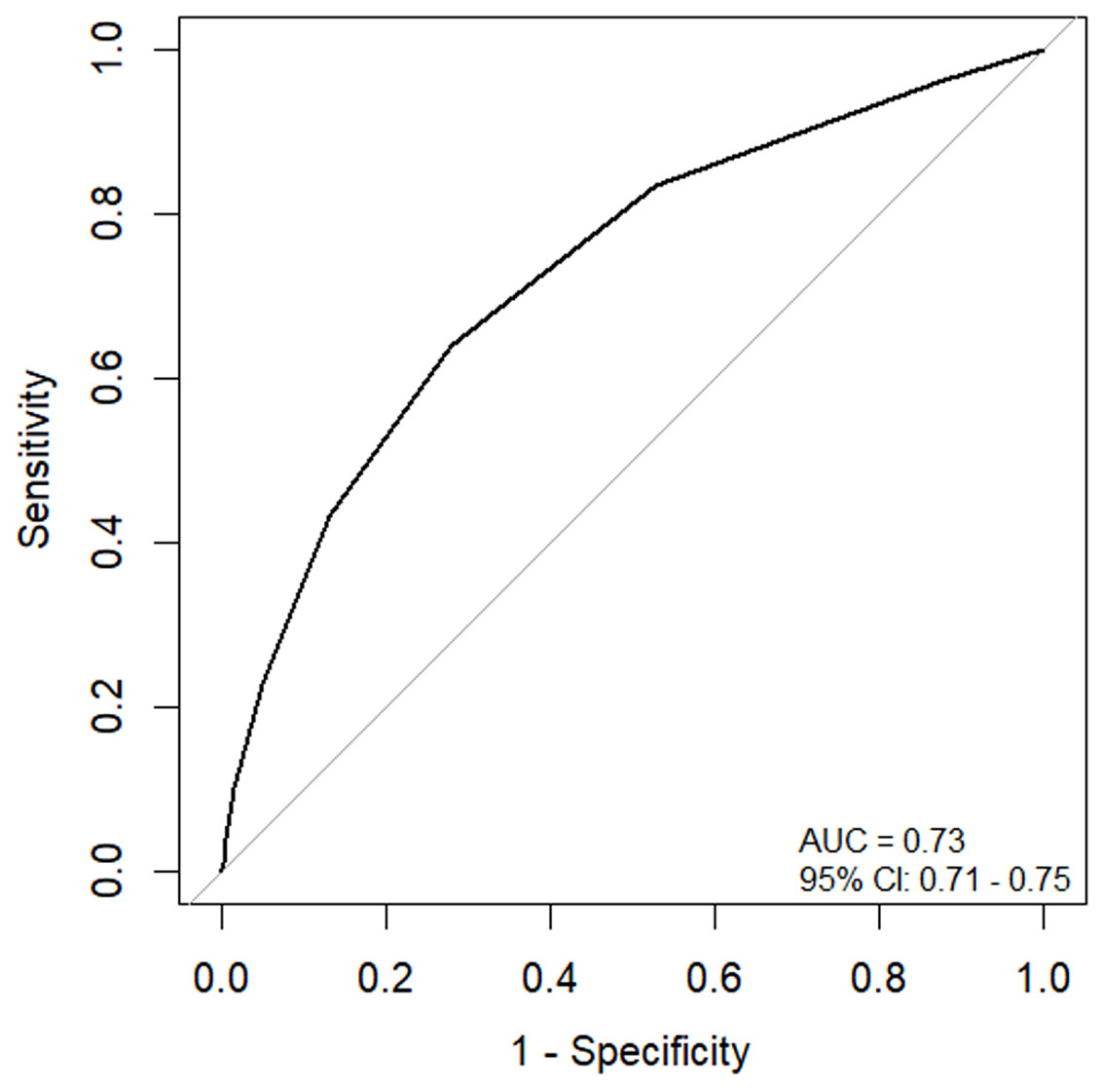
Receiver Operating Characteristic (ROC) curve and corresponding area under the curve of the admission risk score.

Risk scores ranged from 0 to 10 ( Figure 2). We combined score of 8 to 10 because of the small number of patients (26, 0.2%). The majority of the children had a risk score of one (5,153/15,606), while those with higher scores of 8–10 had the least number of children (26/15,606) ( Figure 2). The inpatient mortality rate increased with an increase in the risk score up to a score of 7. Furthermore, we categorized children with scores of zero, 1-4 and ≥ 5 into three categories and assigned them as low risk, moderate risk and high risk, respectively ( Figure 2). Children categorized as low risk (1925, 12.3%) had a mortality rate of 2% (39/1925), moderate risk (12,747, 81.7%) had a mortality rate of 5.6% (710/12,747) and high risk (934, 6.0%) had a mortality rate of 23.4% (219/934). Children with a score of seven had the highest mortality rate (37.2%) ( Figure 2). The risk score had a moderate discriminative performance of identifying children who died and those who didn’t with an AUC of 0.73 (95% CI: 0.71 - 0.75) ( Figure 3).

The sensitivity of the admission risk score decreased from 96% (95% CI: 94.5% – 97.1%) to 3.9% (95% CI: 2.8% – 5.3%) as the cutoff point changed from 1 to 7. In contrast, the specificity increased from 12.9% (95% CI: 12.3% – 13.4%) to 99.5% (95% CI: 99.4% – 99.7%). A cutoff of ≥3 showed a more balanced sensitivity and specificity of 63.8% (95% CI: 60.7%–66.9%) and 72.2% (95% CI: 71.5% – 72.9%), respectively, compared to other cutoff points ( Table 3).

**Table 3.** Performance of the admission risk score in predicting mortality.

	Sensitivity (95% CI)	Specificity (95% CI)	PPV (95% CI)	NPV (95% CI)
**Risk score ≥1**	96.0% (94.5 - 97.1)	12.9% (12.3 - 13.4)	6.8% (6.4 - 7.2)	98% (97.2 - 98.6)
**Risk score ≥2**	83.4% (80.9 - 85.7)	47.3% (46.4 – 48.1)	9.5% (8.8 – 10.1)	97.7% (97.4 – 98.1)
**Risk score ≥3**	63.8% (60.7 - 66.9)	72.2% (71.5 - 72.9)	13.2% (12.2 – 14.2)	96.8% (96.4 - 97.1)
**Risk score ≥4**	43.3% (40.1 - 46.5)	87.0% (86.4 - 87.5)	18.0% (16.5 - 19.6)	95.8% (95.5 - 96.2)
**Risk score ≥5**	22.6% (20.0 - 25.4)	95.1% (94.8 - 95.5)	23.4% (20.8 – 26.3)	94.9% (94.5 - 95.2)
**Risk score ≥6**	10.0% (8.2 -12.1)	98.4% (98.2 – 98.6)	29.8% (24.8 - 35.0)	94.3% (93.9 - 94.7)
**Risk score ≥7**	3.9% (2.8 - 5.3)	99.5% (99.4 - 99.7)	36.5% (27.3 - 46.6)	94.0% (93.6 - 94.4)

### Association between the risk categories and mortality

The association between risk categories and mortality was statistically significant (p < 0.001), as determined by Pearson’s chi-squared test, indicating a strong correlation between higher-risk categories and increased mortality rates.

## Discussion

The initial stage towards broader implementation of clinical risk scores is to validate the current scores in diverse patient populations. In this external validation, we assessed the performance of an admission risk score to predict inpatient paediatric mortality in two public hospitals. The risk score had a moderate discriminatory performance in identifying children at risk of mortality during hospitalization.

The risk score achieved an AUC of 0.73 which was close to that in the derivation cohort (AUC of 0.76) ^14^. This suggests that the risk score moderately distinguishes between children at risk of mortality and those who are not. An AUC value of 0.73, although not indicative of perfect discrimination, still indicates a performance better than chance. This level of accuracy could still be useful in the context of public hospitals in low-resource settings, where advanced diagnostic equipment may be scarce. Our findings demonstrate that the risk score can serve as a valuable complementary tool to clinical judgment, assisting healthcare professionals in making informed decisions regarding patient care.

Compared to other paediatric mortality risk scores, the admission risk score exhibits both similarities and differences in performance and applicability. For instance, the Respiratory Index of Severity in Children (RISC) and RISC-Malawi scores were designed for low-resource settings and have been validated in paediatric populations with acute lower respiratory infections ^25, 26^. The RISC score has demonstrated moderate-to-good performance in different studies, which is comparable to the performance of the admission risk score ^25, 26^. This similarity implies that the admission risk score, like RISC, is moderately effective in predicting mortality, especially in environments where healthcare resources are limited.

In contrast to the admission risk score evaluated in this study, other risk scores such as the paediatric logistic organ dysfunction (PELOD), Pediatric Index of Mortality (PIM) and Pediatric Risk of Mortality (PRISM) scores have demonstrated greater discriminatory power in predicting paediatric mortality ^27– 29^. The PELOD, PIM and PRISM scores, widely utilized in intensive care units (ICUs) globally, comprise a broad array of physiological and diagnostic variables and have shown high discriminatory power, with area under the curve (AUC) values above 0.8 ^27, 30^. In contrast to our risk score, these scores employ detailed clinical and laboratory data that may not be accessible in lower-resource settings. Thus, while risk scores such as PELOD, PIM, and PRISM may offer higher predictive accuracy, the admission risk score remains valuable because of its feasibility and applicability in general paediatric wards, where detailed data may be limited, highlighting the need for context-specific tools that balance precision, logistics, data availability, and practicality.

When patients were classified into low, moderate, and high-risk groups, the mortality rate reflected the assigned category. Children with higher risk scores were more likely to die, thereby validating the usefulness of the risk score in categorising patients based on their probability of mortality. This observation is consistent with prior research that has demonstrated the value of risk scores in paediatric populations, particularly in settings where resources are limited and the burden of disease is high ^14, 31^. By classifying children into specific risk groups, healthcare providers can prioritise care for those in greatest need, potentially enhancing survival rates. This approach aligns with the principles of precision medicine, which aims to tailor medical decisions and treatments to individual patients based on their specific risk profiles and characteristics ^32, 33^.

The admission risk score is a vital tool for improving paediatric care in resource-constrained settings. It equips healthcare providers with a practical method to identify children at higher mortality risk upon hospital admission, facilitating effective prioritization of care and resource allocation. This score is especially valuable in environments with limited healthcare resources, such as insufficient intensive care units and specialized staff. However, despite its performance, several key factors may impede its widespread adoption in low-resource environments. These include healthcare provider acceptance, lack of support from hospital management, implementation complexity, and challenges in integrating the score into existing digital health solutions ^8, 34, 35^. These factors should be carefully considered and addressed when implementing the admission risk score in clinical settings to ensure successful adoption and utilization.

This study has some limitations that should be considered when interpreting the results. Using data from only two public hospitals in Kenya may limit the generalizability of the findings to other settings, particularly those with different healthcare systems or patient populations. Although the CIN database serves as a valuable repository of longitudinal data from multiple public hospitals, its primary purpose is to enhance documentation practices and improve the quality of care for paediatric patients rather than for development and validation of risk scores or prediction models. Consequently, some clinical parameters included in the risk score had missing data. One of these variables was the ability to sit up or stand, which was part of the original score but was not documented in the database. This resulted in a maximum of 12 variables instead of 13. Nonetheless, the performance of the risk score was similar to that of the derivation cohort.

## Conclusion

The admission risk score demonstrated a moderate ability to predict paediatric inpatient mortality. This score can complement clinical evaluations and help prioritize hospitalized children at high risk of mortality. However, further research is needed to assess its performance in other geographical regions with different healthcare systems as well as to understand its practical implications in clinical settings.

## Ethics and consent

The study received ethical approval from the Kijabe Hospital Institutional Scientific and Ethical Review Committee (approval number: KH/ISERC/02718/0011/2024) and the Kenya Medical Research Institute's Scientific and Ethical Review Unit (SERU) for the CIN project (approval number: 3459). Individual consent from the patients to access de-identified clinical data was waived by the ethics committees.

## Data Availability

Harvard Dataverse: Replication Data for: External validation of an admission risk score for predicting inpatient paediatric mortality in two Kenyan public hospitals ( https://doi.org/10.7910/DVN/O0J6DD) This project contains the following underlying data: - Data_2017-2023_csv (Demographic, clinical, medical history and hospital outcome data of participants) - CIN_Dictionary (Data dictionary for the tool used to routinely capture data on inpatient paediatric clinical care) The data utilized in this study are owned by the Kenyan Ministry of Health and County Governments and as the data might be used to de-identify hospitals, the study authors are not permitted to share the source data directly. Users interested in accessing and reusing these data can submit a formal request to the KEMRI Wellcome Trust Research Programme Data Governance Committee (email: dgc@kemri-wellcome.org).
