# Supplementary material for: External validation of an admission risk score for predicting inpatient paediatric mortality in two Kenyan public hospitals
Source: Wellcome Open Res. 2024 Dec 24;9:732. [Version 1] doi: 10.12688/wellcomeopenres.23471.1 (PMC11914872; doi:10.12688/wellcomeopenres.23471.1)
Supplement: Peer review history

# Reviewer report: Reviewer response for version 1

**DOI:** 10.21956/wellcomeopenres.25883.r119836

**Published:** 2025-03-17

**Authors:** Stephen Allen

**Affiliations:** 1 Liverpool School of Tropical Medicine, Liverpool, UK

This is a valuable study that assesses in a comparable setting the predictive value of an existing risk score done on admission that was developed in a low-resource setting. The score is comprised of a limited number of variables all of which should be able to be assessed and documented reliably with appropriate staff training but without additional resources. A significant strength of the study is the use of the extensive CIN database which collects routine data – rather than data collected in the context of a research study. This allows the risk score to be assessed in a “real-life” setting. Relatively few records were excluded because of too much missing data.

 The study included a broad age range (1 month-12 years) with a large number of patients. Since both major causes of mortality and clinical manifestations of illness will vary across this age range, it would have been useful to assess the score in age sub-groups (e.g. infants, 1-4 years, 5-12 years).

 The mortality rates differed between the two hospitals (8.5% vs. 3.3%). It would have been useful to report the average risk scores in each hospital to provide insights into whether there were differences in case-mix or, alternatively, the level of care provided.

 I was unclear why score categories of 0, 1‒4 and ≥5 were selected. From Fig 2, it is difficult to see why score 4 that had a mortality of approx. 14% was not included in the high category.

 As detailed by the authors, a strength of the study is the independent validation of an existing score that was developed in a similar setting. However, the number of variables in the risk score is limited and some potentially important variables were not included in the development of the score in Uganda. For example, given the known increase in mortality associated with severe acute malnutrition, it would be useful to discuss how including malnutrition in the score – even if this was identified by clinical assessment of visible severe wasting and nutritional oedema only (i.e. without calculating z scores). Similarly, given the increasing availability of bedside tests such as blood glucose and O _2 _saturation even low-resource settings, it would have been useful to discuss how these may be incorporated to add value to risk scores.

 Finally, it is clear that identifying children at increased risk of mortality and then taking extra diagnostic, monitoring and/or treatment measures (assuming that these are available) is vital in decreasing mortality in this high-risk group. However, is there a risk of increasing mortality of lower-scoring children if they receive less attention than currently as scoring diverts attention to those at high risk? Even if mortality is increased only slightly in lower-risk children, this may still be important given they are the most numerous by far.

Is the work clearly and accurately presented and does it cite the current literature?

Yes

If applicable, is the statistical analysis and its interpretation appropriate?

Yes

Are all the source data underlying the results available to ensure full reproducibility?

Yes

Is the study design appropriate and is the work technically sound?

Yes

Are the conclusions drawn adequately supported by the results?

Yes

Are sufficient details of methods and analysis provided to allow replication by others?

Yes

Reviewer Expertise:

Global child health; paediatric gastroenterology; malnutrition; clinical trials

I confirm that I have read this submission and believe that I have an appropriate level of expertise to confirm that it is of an acceptable scientific standard.

# Reviewer report: Reviewer response for version 1

**DOI:** 10.21956/wellcomeopenres.25883.r119208

**Published:** 2025-02-25

**Authors:** Thanh Tat Nguyen

**Affiliations:** 1 Woolcock Institute of Medical Research, The Children’s Hospital 2, Ho Chi Minh, Vietnam

**“External validation of an admission risk score for predicting inpatient paediatric mortality in two Kenyan public hospitals”**

 These are my comments in the peer review for the submitted manuscript.

***My general comments:***

 The strength of this study is that the validation of the previously developed risk score included significant clinical predictors of in-hospital death among pediatric patients in resource-limited settings in Africa. These predictors are sensible in terms of pathogenesis and clinical aspects. The rationale for this study is good, and the manuscript has been well written. The sample size of validation cohorts is very large. The analysis is good and acceptable for prognostic validation. However, I think the validation analysis is not robust in terms of assessing only model discrimination. Other important methods of predictive model performance, including calibration (calibration in-the-large, calibration slope, and overall model evaluation), are not presented (hence, I believe this was not performed). The model discrimination showed c-statistics averaging approximately 70% to 73%, which is acceptable.

***These are my specific comments for this manuscript:***

1. I agree with the authors that PELOD-2, PIM, and PRISM scores demonstrated high performance. However, it needs to consider laboratory tests, which are impeded in low-resource countries in Kenya and other African countries. Therefore, a robust clinical prognostic model is required.
2. I believe there should be an additional table describing the causes of death among pediatric patients who died. This will critically aid me and readers to have an insightful cause of patient mortality, cross-matched with the 13 specified clinical predictors of the risk score. These statistics can reveal the most significant cause of death in the region, which helps in designing appropriate proactive interventions. Therefore, the patient mortality rate can be significantly reduced.
3. My further consideration is that pediatric HIV-infected patients commonly present with atypical manifestations. Hence, the symptoms and signs from 13 predictors of risk score may not be present in critically ill HIV-infected patients until the late stage or life-threatening conditions. I raise this point regarding the high prevalence of HIV infections in Africa. Therefore, the risk score may not be sensitive for HIV-infected pediatric population. This might be one possible cause for the lower performance of the predictive model (moderate c-statistics).

***My conclusion:***

 I believe that this manuscript meets the scientific criteria at an acceptable level. I agree this paper to be indexed.

 Many thanks.

Is the work clearly and accurately presented and does it cite the current literature?

Yes

If applicable, is the statistical analysis and its interpretation appropriate?

Yes

Are all the source data underlying the results available to ensure full reproducibility?

Yes

Is the study design appropriate and is the work technically sound?

Yes

Are the conclusions drawn adequately supported by the results?

Yes

Are sufficient details of methods and analysis provided to allow replication by others?

Yes

Reviewer Expertise:

Machine learning, Infectious Disease Modelling, Infectious Diseases, and Pediatrics

I confirm that I have read this submission and believe that I have an appropriate level of expertise to confirm that it is of an acceptable scientific standard.
